# Hypoxia Induces PDK4 Gene Expression through Induction of the Orphan Nuclear Receptor ERRγ

**DOI:** 10.1371/journal.pone.0046324

**Published:** 2012-09-25

**Authors:** Ja Hee Lee, Eun-Jin Kim, Don-Kyu Kim, Ji-Min Lee, Seung Bum Park, In-Kyu Lee, Robert A. Harris, Mi-Ock Lee, Hueng-Sik Choi

**Affiliations:** 1 National Creative Research Initiatives Center for Nuclear Receptor Signals and Hormone Research Center, School of Biological Sciences and Technology, Chonnam National University, Gwangju, Republic of Korea; 2 College of Pharmacy, Seoul National University, Seoul, Republic of Korea; 3 Department of Chemistry, College of Natural Sciences, Seoul National University, Seoul, Republic of Korea; 4 Department of Biophysics and Chemical Biology, College of Natural Sciences, Seoul National University, Seoul, Republic of Korea; 5 World Class University Program, Research Institute of Aging and Metabolism, Kyungpook National University School of Medicine, Daegu, Republic of Korea; 6 Department of Endocrinology and Metabolism, Kyungpook National University Hospital, Daegu, Republic of Korea; 7 World Class University Program, Department of Biochemistry and Molecular Biology, Indiana University School of Medicine and the Roudebush VA Medical Center, Indianapolis, Indiannapolis, United States of America; 8 Research Institute of Medical Sciences, Department of Biomedical Sciences, Chonnam National University Medical School, Gwangju, Republic of Korea; Institut de Génomique Fonctionnelle de Lyon, France

## Abstract

Multiple cellular signaling pathways that control metabolism and survival are activated when cell are incubated under hypoxic conditions. Activation of the hypoxia inducible factor (HIF)-1 promotes expression of genes that increase the capacity to cope with the stress imposed by a reduced oxygen environment. Here we show that the orphan nuclear receptor estrogen related receptor γ (ERRγ) plays a critical role in hypoxia–mediated activation of pyruvate dehydrogenase kinase 4 (PDK4) gene expression. ERRγ mRNA and protein levels were increased by hypoxia or desferrioxamine (DFO) treatment in hepatoma cell lines. Co-expression of HIF-1α and β increased ERRγ promoter activity as well as mRNA expression, while knockdown of endogenous HIF-1α reduced the hypoxia-mediated induction of ERRγ. In addition, hypoxia also increased the promoter activity and mRNA level of PDK4 in HepG2 cells. Adenovirus mediated-overexpression of ERRγ specifically increased PDK4 gene expression, while ablation of endogenous ERRγ significantly decreased hypoxia-mediated induction of PDK4 gene expression. Finally, GSK5182, an inverse agonist of ERRγ, strongly inhibited the hypoxia-mediated induction of PDK4 protein and promoter activity. Regulation of the transcriptional activity of ERRγ may provide a therapeutic approach for the regulation of PDK4 gene expression under hypoxia.

## Introduction

Hypoxia is a pathological state in which cells of the body are not afforded an adequate supply of oxygen. The stress imposed by hypoxic induces expression of a number of genes that are involved in the regulation of respiration, metabolism, and cell survival. Cells under normal oxygen condition convert glucose to pyruvate which enters the mitochondria for further catabolism through the tricarboxylic acid (TCA). This results in ATP production and terminates in the donation of electrons to oxygen. In low oxygen conditions, activation of the hypoxia-inducible factor-1α (HIF-1α) promotes ATP production by increasing the enzymatic capacity for anaerobic glycolysis to compensate for the reduced rate of oxidative phosphorylation [1]. HIF-1 is a heterodimeric transcription factor consisting of an α-subunit and a β-subunit. Stability of the α-subunit is greatly increased under hypoxic conditions. The β subunit, in contrast, is constitutively expressed under both normoxia and hypoxia conditions. The stabilization of HIF-1α in hypoxic cells allows its nuclear translocation and formation of an HIF-1α/β heterodimer [2]. The complex of HIF-1α and β binds to hypoxia response element (HRE; typical HRE sequence is RCGTG) and transactivates a wide variety of genes involved in the hypoxia response such as erythropoietin which induces red blood cell production, vascular endothelial growth factor (VEGF) which promotes angiogenesis, and GLUT1 which increases the efficiency of the glucose uptake [3]. Recent reports have shown that HIF-1α is involved in obesity-related metabolic dysfunction. In early stage of obesity, the elevated expression of HIF-1α is associated with fibrosis and insulin resistance in white adipose tissue [4]. In addition, adipose tissue specific expression of HIF-1α induces impaired energy expenditure and glucose intolerance in brown adipose tissue [5]. These observations highlight the needs for further study of HIF-1-related metabolism disorders.

Estrogen-related receptor (ERRα, ERRβ, and ERRγ) are constitutively active nuclear receptor that contain high levels of sequence identity to estrogen receptors [6]. ERRs bind to classic estrogen response elements (ERE) as dimers, or to extended half-site core sequences (TNAAGGTCA) as monomers [7]. Although ERRs are constitutively active in the absence of endogenous ligands, synthetic compounds that stimulate or inhibit the transcriptional activity of the ERRs have been found [8]. ERRα and γ isoforms are ubiquitously expressed with especially high levels found in the heart, kidney, intestinal tract, skeletal muscle, and brown adipose tissue. In contrast, ERRβ expression is restricted to the brain, kidney and heart. ERRs regulate a number of genes involved in energy homeostasis, cell proliferation and cancer metabolism [6], [9]. Targets of ERRγ include peroxisome proliferator-activated receptor γ coactivator-1α (PGC-1α), pyruvate dehydrogenase kinase (PDK), RARα, cyclin-dependent kinase inhibitors p21 (WAF1/CIP1) and p27 (KIP1). The transcriptional activity of ERRγ relies on interaction with coactivators and corepressors. Indeed, the corepressor known as “small heterodimer partner interacting leucine zipper protein” (SMILE) down-regulates the expression of PDK4 via direct interaction with ERRγ [10]. In addition, GSK5182, a derivative of hydroxytamoxifen (4-OHT), acts as inverse agonist to deactivate ERRγ [11], [12], [13].

PDK isoforms identified in mammals are designated PDK1, PDK2, PDK3, and PDK4. PDK2 and PDK4 isoforms are highly expressed in the liver, heart, and skeletal muscle [14]. By phosphorylation, pyruvate dehydrogenase kinases (PDKs) negatively regulate PDH activity, a key enzyme catalyzing the conversion of pyruvate to acetyl-CoA [15]. HIF-1 directly inactivates the pyruvate dehydrogenase (PDH) through upregulation of pyruvate dehydrogenase kinase 1 (PDK1), which reduces the supply of glucose carbon to the TCA cycle [16]. In particular, PDK1 is involved in adaptation of hypoxia by reducing oxygen consumption and restricting the entry of glycolytic intermediates into the TCA cycle. Recent reports have shown that the PDK1 and PDK3 are HIF-1 target genes. Forced PDK1 expression in HIF-1 null cells increases ATP levels, attenuates hypoxic ROS generation, and rescues these cells from hypoxia-induced apoptosis [17].

In this study, we have shown that ERRγ is required for HIF-1α mediated regulation of PDK4 expression. In addition, we have observed that ERRγ induced PDK4 gene expression under hypoxic by directly binding to the PDK4 promoter. Moreover, the ERRγ specific inverse agonist, GSK5182, reduces hypoxia-induced expression of PDK4. Overall, our observations suggest a critical role for ERRγ in the up-regulation of PDK4 by HIF-1. The findings also suggest that regulation of ERRγ by GSK5182 has therapeutic potential.

## Results

### Hypoxia induces the ERRγ gene expression in liver cell lines

**Figure 1:**
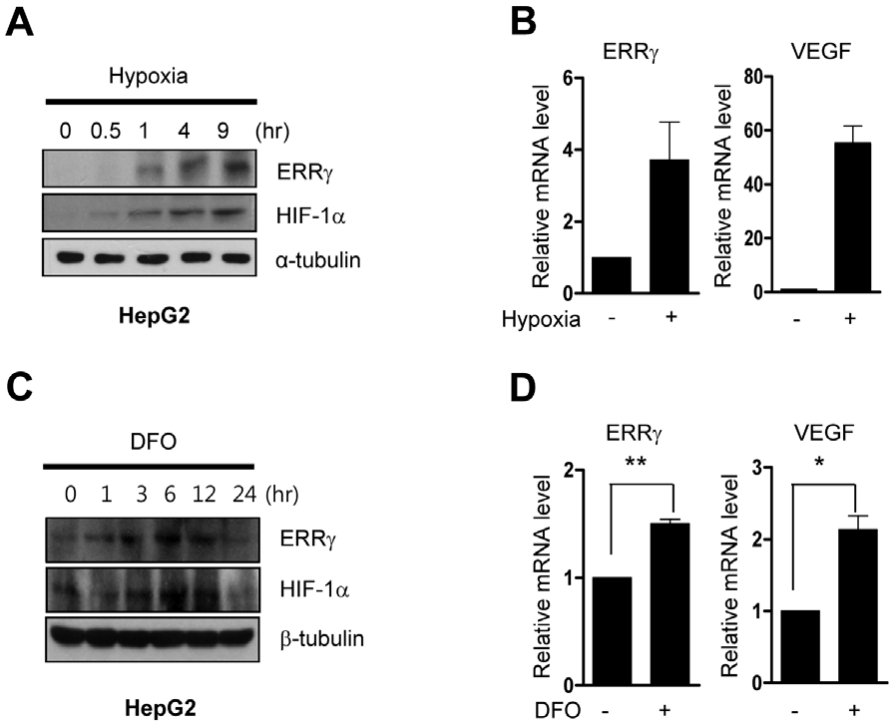
Hypoxia induces the ERRγ gene expression in hepatoma cell lines. (**A–B**), HepG2 cells were seeded in 60-cm^2^ dishes and incubated overnight. Then cells were incubated under hypoxia at indicated time period. The expression of ERRγ was analyzed by Western blot (**A**) and Q-PCR (**B**) analysis. (**C–D**) HepG2 cells were seeded in 60-cm^2^ dishes and incubated overnight and then treated with DFO at indicated concentration and time period. The expression of ERRs was analyzed by Western blot (**C**) and Q-PCR (**D**) analysis. ERRγ gene expression was normalized to L32 gene expression, and α or β-tubulin expression. All data are representative of at least three independent experiments. Error bars show ± S.E.M. ^*^ *P*<0.05, ^**^ *P*<0.01 by two-tailed Student *t*-test.

To our knowledge, no evidence exists for a role for ERRγ in the compensatory response of the liver to hypoxia. To evaluate whether hypoxic conditions affect the expression of ERRγ, HepG2 cells were incubated in the hypoxia chamber for various lengths of time prior to the preparation of RNA and protein extracts. The protein level of ERRγ, as determined by Western blotting, was increased via stimulation of hypoxia in a time dependent manner (Figure 1A). The mRNA level of ERRγ, as determined by quantitative real time PCR (Q-PCR), was likewise increased more than threefold after 9 hr of exposure of the cells to hypoxia (Figure 1B). Hypoxia was confirmed by increased VEGF mRNA and HIF1α protein levels. Moreover, treatment with desferrioxamine (DFO), an iron chelator that interferes with synthesis of cytochromes, increased ERRγ mRNA and protein levels. The protein level of ERRγ began to increase in the first hour of DFO treatment and was maximally increased after 6 hr (Figure 1C). Up regulation of mRNA levels of ERRγ and VEGF were observed after 6 hr of DFO treatment (Figure 1D). Similar results were obtained in mouse hepatoma cell line AML-12 and rat hepatoma cell line H4IIE (data not shown). These findings establish that ERRγ is induced significantly by hypoxia.

### HIF-1α mediates the induction of ERRγ under hypoxic condition

**Figure 2:**
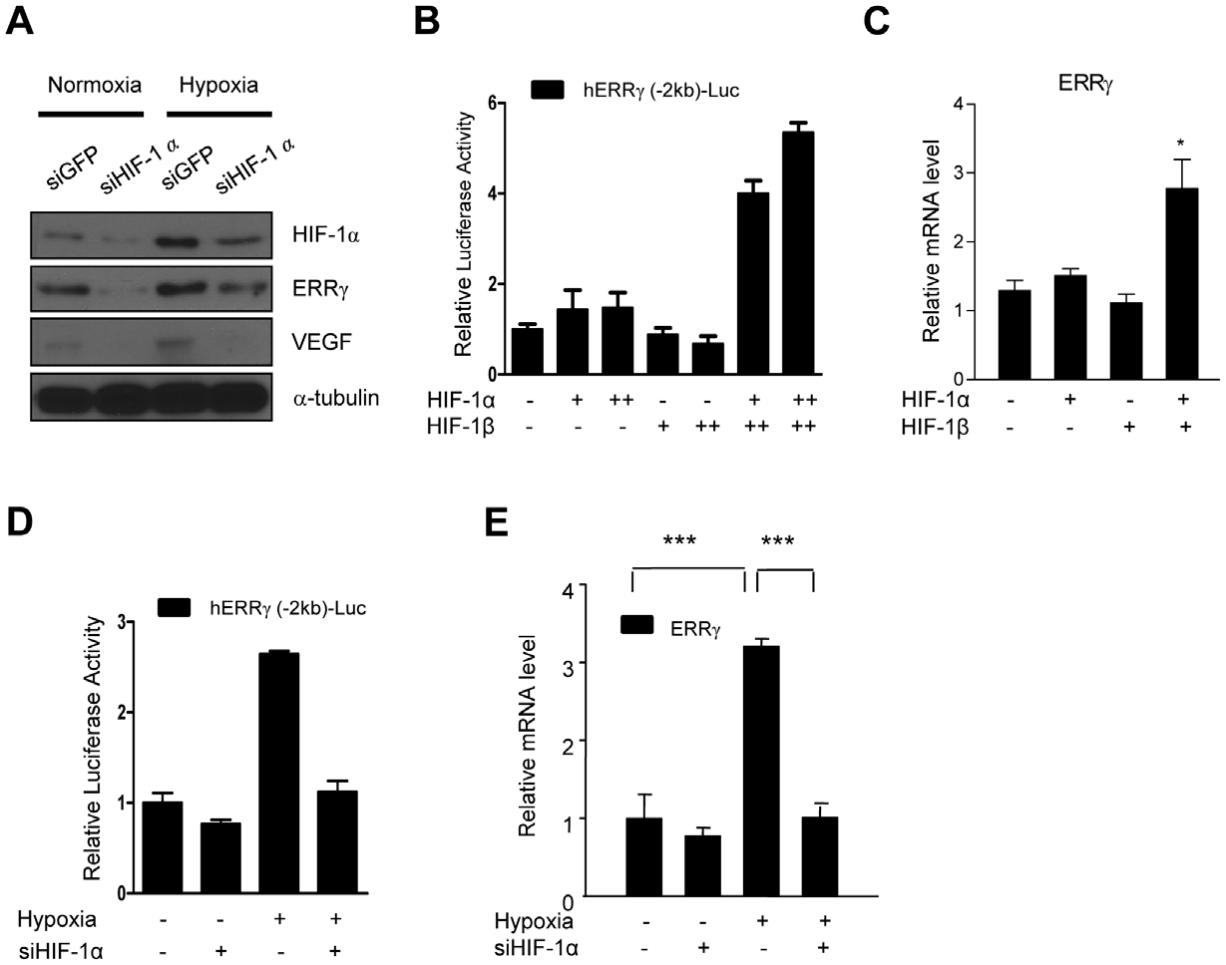
HIF-1α increases the expression of ERR γ. (**A**) HepG2 cells were transfected with nonspecific siRNA (NS) or si-HIF-1α, and isolated total protein was analyzed by Western blot. α-tubulin was used as a control. (**B**) HepG2 cells were transfected with pcDNA3-HIF-1α, pcDNA3-ARNT and hERRγ-Luc, respectively. Experiments were conducted in duplicate and data are expressed as the fold activation relative to the control. (**C**) HepG2 cells were transfected with pcDNA3-HIF-1α and pcDNA3-ARNT and Q-PCR was performed using isolated total RNA. (**D–E**) HepG2 cells were transfected with nonspecific siRNA (NS) or si-HIF-1α. After transfection, lysates were utilized for luciferase and β-galactosidase assay (D). Q-PCR was performed using isolated total RNA from HepG2 cells (E). All data are representative of at least three independent experiments. Error bars show ± S.E.M. ^*^ *P*<0.05, ^***^ *P*<0.001 by two-tailed Student *t*-test.

**Figure 3:**
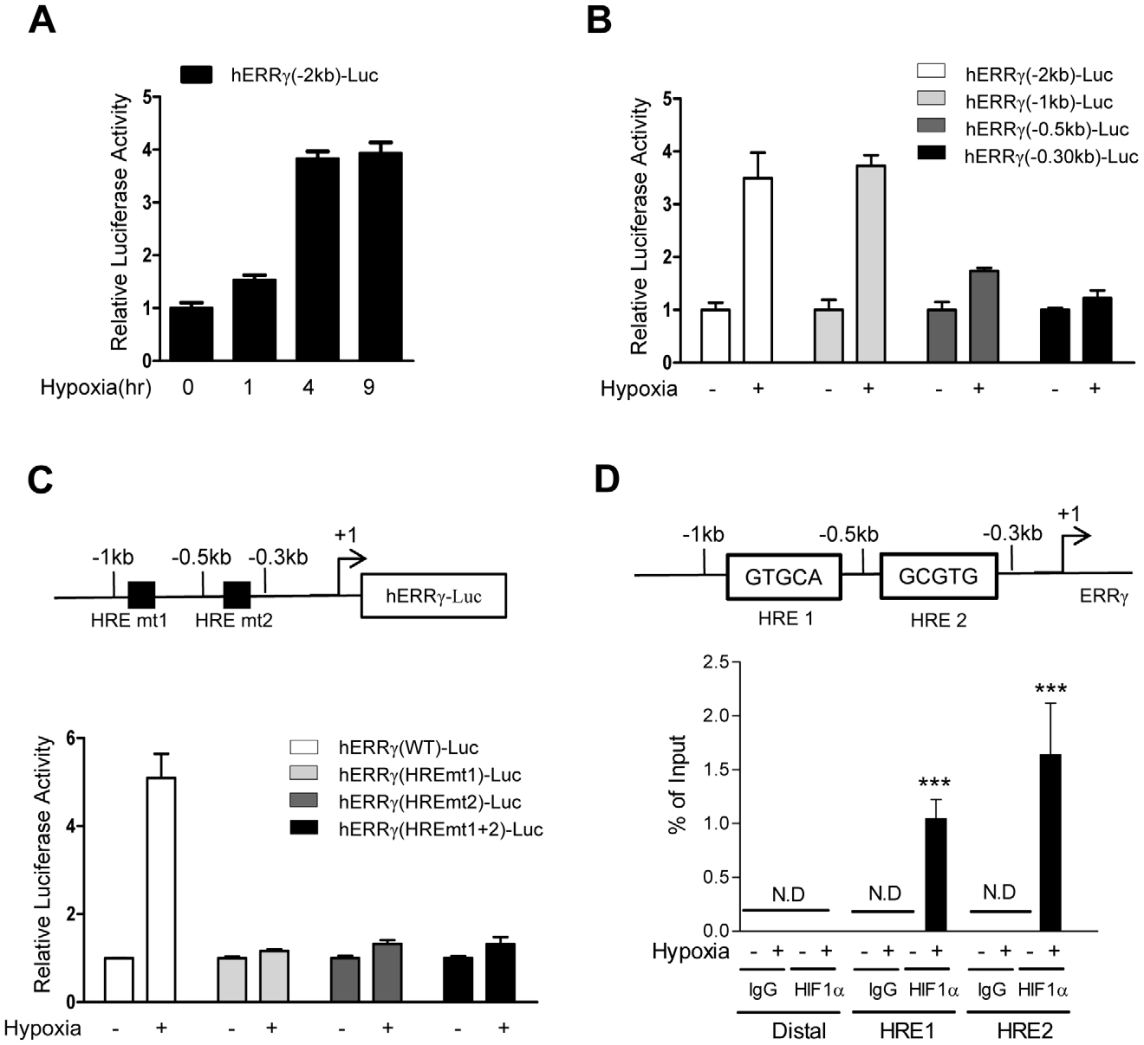
Hypoxic activation of HIF-1α directly regulates the transcriptional activity of ERRγ. (**A**) HepG2 cells were transfected with hERRγ-Luc. After 24 h of the transfection, HepG2 cells were exposed to hypoxia for indicated time period. Experiments were carried out in triplicate and data are expressed as the fold activation relative to the control. (**B–C**) HepG2 cells were transfected with hERRγ (−2 kb)-Luc, hERRγ (−1 kb)-Luc, hERRγ (−0.5 kb)-Luc, hERRγ (−0.3 kb)-Luc, hERRγ (HREmt1)-Luc, hERRγ (HREmt2)-Luc, hERRγ (HREmt1+2)-Luc. After 24 h of the transfection, HepG2 cells were exposed to hypoxia for 9 hr and analyzed using luciferase and β-galactosidase assay. Experiments were performed in duplicate and data are expressed as the fold activation relative to the control. (**D**) ChIP assay: HepG2 cell was exposed to hypoxia for 9 hr. Input represents 10% of purified DNA in each sample. Cell extracts were immunoprecipitated with anti-HIF-1α and purified DNA samples were employed for Q-PCR with primers binding to HRE1 (−1080 to −849) and HRE2 (−508 to −295) and distal site (−1826 to −1586) on the *ERRγ* gene promoter. All data are representative of at least three independent experiments. Error bars show ± S.E.M. ^***^ *P*<0.001 by two-tailed Student *t*-test.

To confirm whether hypoxia signaling up-regulates the expression of ERRγ via HIF-1, we examined whether knockdown of HIF-1α affected the expression of ERRγ in hypoxia. Knockdown of HIF-1α under both normoxia and hypoxia led to significant decrease in protein levels of ERRγ (Figure 2A). Co-transfection of HIF-1α with HIF-1β increased the luciferase activity of human ERRγ promoter (Figure 2B). In addition, the mRNA expression of ERRγ was also induced by HIF-1α and β over-expression (Figure 2C). However, knockdown of HIF-1α dramatically reduced hypoxia-induced activation of ERRγ promoter and the mRNA expression of ERRγ (Figure 2D and E). These results suggest that the transcription of ERRγ gene is directly regulated by HIF-1. To establish the mechanism responsible for the induction of ERRγ expression under hypoxic conditions, transient transfection assays using a human ERRγ promoter (−2 kb)/luciferase reporter construct were performed in HepG2 cells. The luciferase activity of the construct was increased by incubation of the cells under hypoxic condition (Figure 3A). A search of the promoter region of the ERRγ gene for the consensus HIF-1 binding site (5′-(A/G)CGTG-3′; [18]) revealed two putative HRE consensus sequences on human ERRγ promoter. To identify the HRE sequence motif, we generate serial deletion constructs of hERRγ promoter. The −2 kb and −1 kb forms of the ERRγ promoter/reporter construct exhibited the same luciferase activity under hypoxic conditions. In contrast, most of the response to hypoxia was lost in the −0.5 and −0.3 kb forms of the promoter (Figure 3B), consistent with the locations of the putative HRE binding sites (Figure 3C). Mutation of the putative HRE binding sites led to significant reduction of promoter activity of ERRγ (Figure 3C). The functional importance of the sites was further confirmed by ChIP assay. High levels of HIF-1α were found associated with HRE1 and HRE2 on the ERRγ promoter under hypoxic conditions (Figure 3D). These results indicate that hypoxia directly regulates the expression of ERRγ via HIF-1α.

### Hypoxia increases the PDK4 gene expression via ERRγ

**Figure 4:**
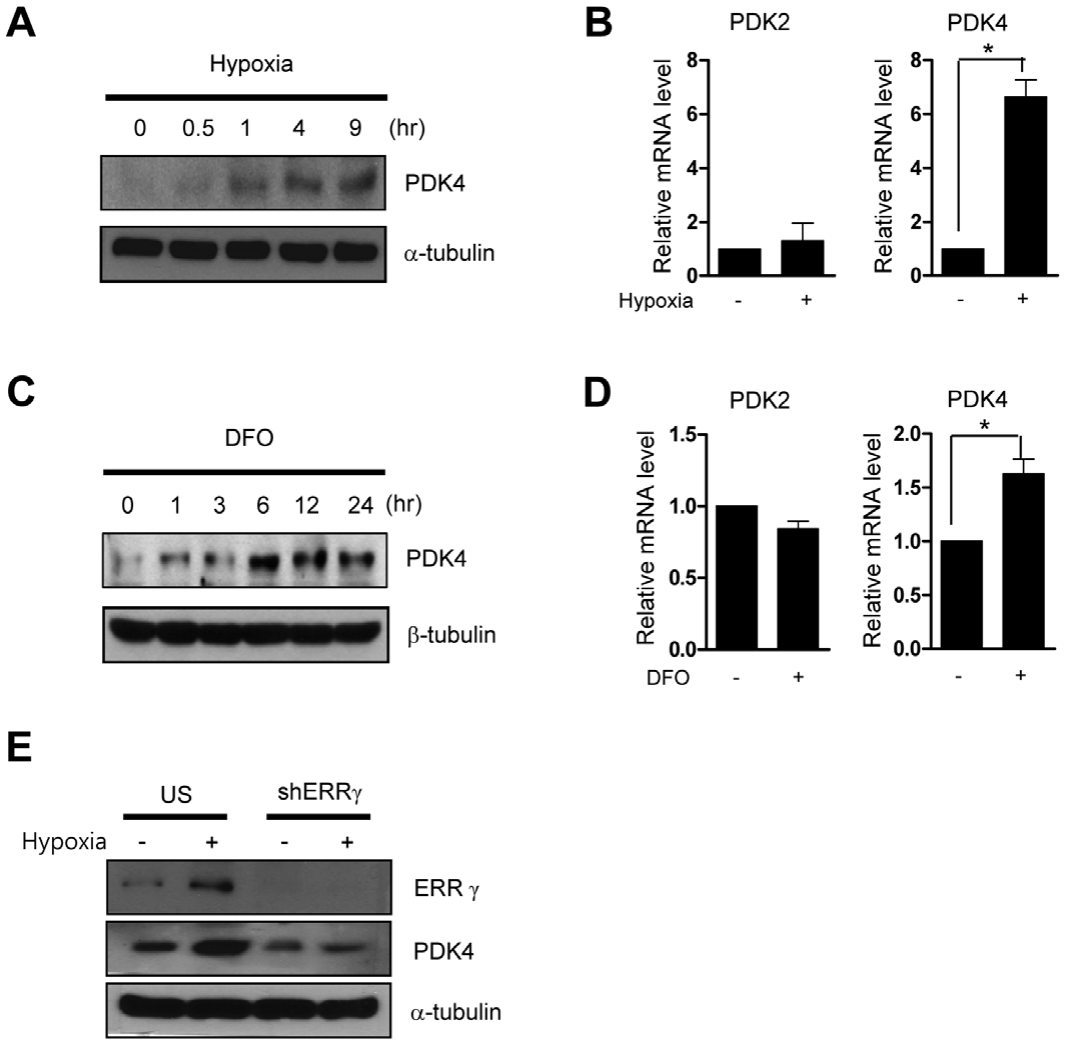
ERRγ mediates the hypoxia induced expression of PDK4. (**A–B**) HepG2 cells were seeded in 60-cm^2^ dishes and exposed to hypoxia for indicated time period. Total RNA and protein were isolated and used for Western blot (**A**) and Q-PCR (**B**), respectively. (**C–D**) HepG2 was treated with DFO for indicated concentration and time period and then cells were harvested. Total protein was harvested for Western blot (**C**) using indicated antibodies and was normalized to β-tubulin expression. And total RNA was isolated for Q-PCR (**D**). The mRNA levels of PDK2 and PDK4 were normalized to L32 gene expression. (**E**) Effect of knockdown of ERRγ. HepG2 cells were infected with Ad-US and Ad- shERRγ for 48 hr, respectively. Total protein was isolated for Western blot analysis of PDK4 and then was normalized to β-tubulin or α-tubulin expression. All data are representative of at least three independent experiments. Error bars show ± S.E.M. ^*^ *P*<0.05 by two-tailed Student *t*-test.

It is well established that hypoxia regulates glycolysis and mitochondrial respiration through up-regulation of PDK1 via HIF-1α in human breast and renal cancer cell lines [16], [17]. We examined whether PDK2 and PDK4, which are dominantly expressed in liver, was induced by hypoxia in HepG2 cells. PDK4 protein was marginally increased at 1 hr and dramatically increased at 4 hr by hypoxia (Figure 4A). In addition, the mRNA level of PDK4 but not PDK2 was increased by almost five fold by hypoxia (Figure 4B). Consistent with above results, DFO treatment also increased the mRNA and protein levels of PDK4 but not PDK2 in HepG2 cells (Figure 4C–D). To confirm the regulation of PDK4 by ERRγ, we examined the expression of PDK2 and PDK4 after overexpression of ERRγ in HepG2 cells. The mRNA level of PDK4 was significantly increased but the mRNA level of PDK2 was not changed (data not shown). To further confirm whether hypoxia up-regulates the expression of PDK4 via ERRγ, we examined whether hypoxia-induced expression of PDK4 was affected by knockdown of ERRγ. Adenovirus-mediated knockdown of ERRγ significantly reduced the protein levels of PDK4 with or without hypoxia (Figure 4E).These results demonstrate that hypoxia regulates the expression of PDK4 via ERRγ.

### ERRγ directly regulates hypoxia induced PDK4 transcription

**Figure 5:**
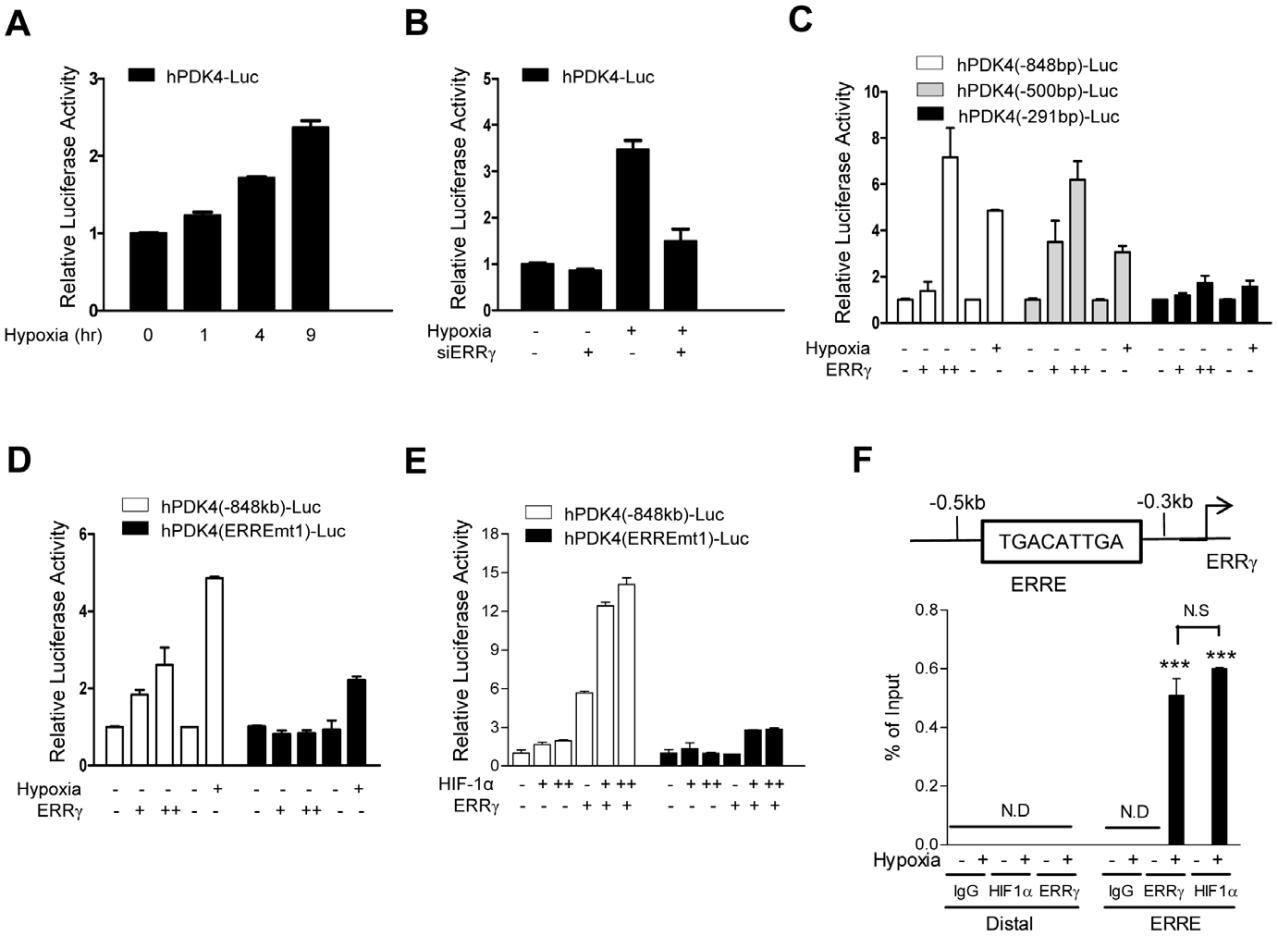
ERRγ directly regulates hypoxia mediated PDK4 gene expression. (**A**) HepG2 cells were transfected with hPDK4-Luc. After transfection, the cells were exposed to hypoxia for indicated period and lysates were utilized for luciferase and β-galactosidase assay. Experiments were done in triplicate and data are expressed as the fold activation relative to the control. (**B**) HepG2 cells were seeded in 60-cm^2^ dishes and trasnfected siERRγ and control siRNA for 72 hr and then exposed hypoxia for 9 hr. Cells were harvested for analyzing luciferase and β-galactodidase assay. (**C–D**) HepG2 cells were transfected with several deletion constructs of hPDK4 (−848)-Luc, hPDK4 (−500)-Luc, hPDK4 (−291)-Luc and hPDK4-mtERRE1-Luc with pcDNA3-ERRγ in the presence or absence with hypoxia exposure, respectively. 48 hr after transfection, the cells were harvested and performed luciferase and β-galactodidase assay. Experiments were done in duplicate and data expressed as the fold activation related to control. (**E**) HepG2 cell were transiently transfected with hPDK4 (−848)-Luc, hPDK4-mtERRE1-Luc, pcDNA3-ERRγ and pcDNA3-HIF-1α. (**F**) ChIP assay: HepG2 cell was exposed to hypoxia for 9 hr. Input represents 10% of purified DNA in each sample. Cell extracts were immunoprecipitated with anti-ERRα and purified DNA samples were employed for Q-PCR with primers binding to ERRE (−502 to −252) and distal site (−1056 to −886) on the *PDK4* gene promoter. All data are representative of at least three independent experiments. Error bars show ± S.E.M. ^***^ *P*<0.001 by two-tailed Student *t*-test.

To confirm the induction of PDK4 by hypoxia, HepG2 cells transfected with human PDK4 promoter-luciferase reporter construct were incubated in the hypoxia chamber in a time study. As shown in Figure 5A, PDK4 promoter activity was increased in hypoxic condition. To examine whether ERRγ mediates hypoxia-induced PDK4 expression, we examined the promoter activity of PDK4 after siRNA-mediated knockdown of ERRγ. As expected, knockdown of ERRγ reduced the response of the PDK4 promoter to hypoxia (Figure 5B). A highly conserved binding site for the α isoform of ERR (TGACATT; bp −371 to −363) on the PDK4 promoter has been defined in previous studies by others [19]. To examine whether ERRγ mediates the hypoxia-induced expression of PDK4 via this HRE, we designed constructs mutated (hPDK4(ERREmt1)-luc) and deleted (hPDK4(−500 bp)-luc) and (hPDK4(−291 bp)-luc). After transfection of deleted and point mutated constructs, ERRγ-mediated promoter activity of PDK4 was measured. As expected, hypoxia increased the luciferase activity of hPDK4(−848 bp)-luc and hPDK4(−500 bp)-luc. However, this effect was abolished in the hPDK4(−291 bp)-luc and also in the ERRE mutated construct (Figure 5C–D). It has been reported that HIF1α directly interacts with ERRγ, and potentiates the transcriptional activity of ERRγ during hypoxia stimulation [20]. We examined the effect of HIF1α on ERRγ-mediated PDK4 promoter activity using transient transfection in HepG2 cells. Indeed, ERRγ-mediated PDK4 promoter activity was markedly potentiated by co-transfection of HIF1α, while mutation of the PDK4 ERRE abolished the effect of ERRγ and HIF1α (Figure 5E). Moreover, HIF1α could not activate PDK4 promoter in the absence of ERRγ or PDK4 ERRE. Next, we performed chip assay to confirm the hypoxia treated regulation of ERRγ on PDK4 promoter. We observed that ERRγ recruitment on PDK4 promoter was increased when the cell were rendered hypoxic for 9 hr and that HIF-1α was recruited to PDK4 ERRE and the magnitude was similar with that of ERRγ (Figure 5F), suggesting a two- pronged regulation of HIF1α for ERRγ-mediated PDK4 gene expression during hypoxia condition: HIF1α increases ERRγ gene expression, and enhances transcriptional activity of ERRγ for PKD4 gene transcription. Overall, these results indicate that ERRγ directly regulates hypoxia-induced PDK4 transcription in a HIF-1α-dependent manner.

### ERRγ inverse agonist GSK5182 down-regulates the hypoxia-induced PDK4 expression

**Figure 6:**
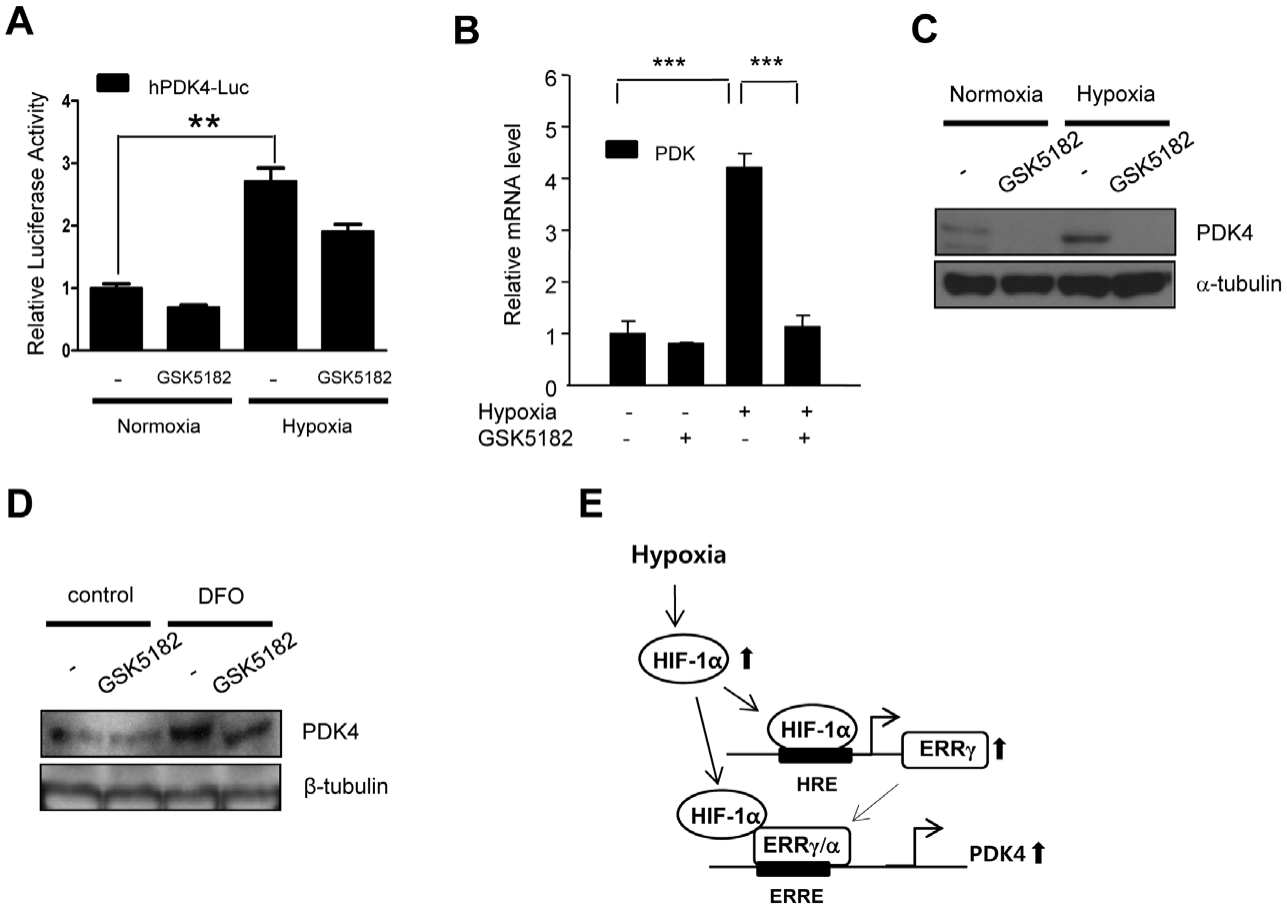
ERRγ inverse agonist GSK5182 down-regulates the hypoxia-induced PDK4 expression. (**A–B**) HepG2 cells were transfected with hPDK4-Luc. After transfection, the cells were exposed in hypoxia and treated with or without GSK5182. Harvested lysates were utilized for luciferase and β-galactosidase assay (A). Q-PCR was performed using isolated total RNA (B). Experiments were done in triplicate and data are expressed as the fold activation relative to the control. (**C**) HepG2 cells were seeded in 60-cm^2^ dishes and incubated overnight. The cells were incubated in hypoxia and treated with or without GSK5182. Total protein was harvesed for Western blot analysis using indicated antibodies. (**D**) HepG2 cells were seeded in 60-cm^2^ dishes and incubated overnight. The cells were treated with or without chemicals (DFO and GSK5182) for 6 hr. Total protein and mRNA were isolated for Western blot assay and RT-PCR and normalized with α or β-tubulin and β-actin. (**E**) A schematic representation. All data are representative of at least three independent experiments. Error bars show ± S.E.M. ^**^ *P*<0.01, ^***^ *P*<0.001 by two-tailed Student *t*-test.

Next, we used GSK5182, an ERRγ specific inverse agonist, to determine its effect on PDK4 expression. Our previous work has shown that SMILE forms an inhibitory complex with ERRγ on the PDK4 promoter and that the recruitment of SMILE is increase by the ERRγ specific inverse agonist GSK5182 [10]. To test whether hypoxia-mediated induction of PDK4 is regulated by GSK5182, we measured the luciferase activity of human PDK4 promoter after exposing cells to hypoxia and treating with GSK5182. As shown in Figure 6A and B, the promoter activity and mRNA levels of PDK4 were reduced under both normoxia and hypoxia by GSK5182. To further confirm down-regulation of PDK4 expression by GSK5182, we tested changes in expression of PDK4 after GSK5182 treatment in hypoxia-treated HepG2 cells. As expected, the treatment of GSK5182 repressed the expression of PDK4 under hypoxic condition and normoxic condition (Figure 6C). Consistent with above data, pre-treatment with GSK5182 also repressed DFO-induced expression of PDK4 (Figure 6D).These results suggest that ERRγ inverse agonist GSK5182 strongly suppresses hypoxia-induced activation of PDK4.

## Discussion

Cells under the hypoxic stress induce several adaptive responses that increase the rate of glycolysis and angiogenesis and reduce mitochondrial respiration. HIF-1 plays a critical role in hypoxia-mediated change in the expression of genes involved in these adaptive responses. Several reports have also shown that hypoxia regulates expression of the orphan nuclear receptor ERRα through an interaction with PGC-1α that occurs independently of HIF-1. Indeed, VEGF is directly up-regulated by ERRα under low oxygen concentration through a cooperative interaction with PGC-1a that occurs independently of the HIF pathway in muscle [21]. In the current study, we observed that hypoxia increased the expression of ERRγ (Figure 1), whereas siRNA-mediated knockdown of HIF-1α blocked hypoxia induced expression of ERRγ (Figure 2A). Our results show that induction of ERRγ by hypoxia is dependent upon HIF-1α. Interestingly, ERRγ and HIF-1α protein levels were decreased after 24 hr-treatment with DFO (Figure 1C). Although HIF-1α has been to be generally accepted as a pro-survival factor, it is able to induce apoptosis during severe (<0.2%) or prolonged (>24 hr) hypoxia. Hypoxia-induced apoptosis is indeed most common under these severe conditions [22]. DFO, a hypoxia-mimetic agent, functions as an iron chelator and has been utilized in hypoxic study. In addition, it is a well known activator of HIF-1, suggesting that it is also able to induce apoptosis during severe or prolonged DFO exposure, similar with that of hypoxia. Indeed, the magnitude of DFO-mediated induction of HIF-1α protein levels was higher than that of hypoxia (Figure 1C). Therefore, the reduction of HIF-1α and ERRγ protein levels in HepG2 cells after 24 hr-treatment with DFO is able to be caused by cell apoptosis, which depends on multiple factors such as cell type and the level or duration of DFO-mediated hypoxia.

Previous reports have established that ERRβ is a positive and essential component of the HIF transcriptional complexes that control hypoxia inducible gene expression. ERRα and ERRβ serve as essential cofactors of HIF in mediating the hypoxic response. ERRs recognize the functional HIF heterodimers but do not bind to single HIF-1α or HIF-1β. Both ERRα and ERRβ stimulate the expression of HIF-1 target genes such as erythropoietin, the key angiogenic factor VEGF, and the glycolytic enzyme PGK-1 in human cancer cell lines [20]. However, it has not established that HIF-1α has an effect upon the transcription of ERRs. In the present study, promoter activity and mRNA expression of ERRγ was increased under hypoxia condition and reduced by siRNA-mediated knockdown of HIF-1α (Figure 2D and E). Our study indicates that HIF-1α plays an essential role in the regulation of ERRγ under hypoxia condition. Moreover, we found two putative binding site of HIF-1α on the ERRγ promoter by promoter mapping (Figure 3B). Furthermore, promoter activity in response to hypoxic was abolished in these site-mutated constructs (Figure 3C). And finally, ERRγ regulation of HIF-1α under hypoxia condition was confirmed by ChIP assays (Figure 3D). These results show that HIF-1α directly regulates the transcription of ERRγ under hypoxia.

It is well known that HIF strongly reduces oxidative glucose metabolism via induction of PDKs which blocks conversion of pyruvate to acetyl CoA which accelerates the production of ATP by anaerobic glycolysis [17]. The pyruvate dehydrogenase complex (PDC) is a key enzyme catalyzing the conversion of pyruvate to acetyl CoA [15]. PDKs negatively regulate PDC activity via phosphorylation of PDC. HIF-1α binds directly to the PDK1 promoter and activates the transcription of PDK1 in human renal cell carcinoma cell lines. In response to hypoxia, PDK1 increases dramatically in HIF-1α^+/+^ MEFs but not in HIF-1α^−/−^ MEFs [16], [17]. Indirect evidence that HIF-1α also regulates PDK4 expression was presented in a previously study with SIRT6 deficient mice [23]. The histone deacetylase SIRT6 regulates glucose homeostasis by suppressing the activity of HIF-1α. By increasing HIF-1α, SIRT6 deficiency results not only in increased expression of PDK1 and multiple glycolytic genes but also in increased expression of PDK4 [23]. In the current study, hypoxia specifically increased PDK4 but not PDK2 in hepatoma cell lines. Adenovirus-induced overexpression of ERRγ increased the expression of PDK4 (data not shown). Moreover, ablation of endogenous ERRγ abolished hypoxia-induced expression of PDK4. Transfection assays of deleted and mutated PDK4 promoter constructs showed that ERRγ directly regulates in the hypoxia induced transcription of PDK4. The ChIP assay showed that the recruitment of ERRγ on the PDK4 promoter was significantly increased by exposure of hypoxia. These results demonstrate that ERRγ directly regulates the transcription of PDK4 and plays an important role in regulation of PDK4 expression under hypoxia.

On the other hand, we found that both basal and hypoxia-mediated induction of PDK4 protein was not completely abolished by knockdown of ERRγ (Figure 4E), and that ablation of ERRγ in HepG2 cells did not block absolutely basal and hypoxia-induced activity of PDK4 promoter (Figure 5B), suggesting the establishment of a compensatory transcriptional control mechanism of ERRα in the absence of ERRγ. It has been reported that the transcriptional activity of three ERRs, which are constitutively active transcription factor in the absence of endogenous ligands, depends on nuclear receptor coregulators, such as steroid receptor coactivator 2 (SRC-2), PGC-1, receptor interacting protein 140 (RIP140) and small heterodimer partner (SHP). In addition, the ERRs can bind to extended half-site core sequences as either monomers or dimmers. Although their functions in terms of the transcriptional output of each ERR are somewhat complicated, they can regulate the same direct target genes [6]. Consistent with these reports, it has been reported that both ERRα and γ could increase the PDK4 gene expression in hepatoma cell lines [19]. Moreover, both ERRα and γ physically interact with HIF-1α, and mediate HIF-1α-induced transcription during hypoxia stimulation [20]. These reports are further supported by the previous report that ERRα and ERRγ could directly control the same target genes in cardiomyocytes, and ERRγ can bind the DNA in the absence of ERRα [24]. Therefore, the molecular mechanism causing different transcriptional regulation or output of each ERR isoform for downstream targets requires further characterization.

Previously, we reported that the binding of the ERRγ specific inverse agonist GSK5182 to ERRγ recruits corepressor SMILE-SIRT complex, which leads to the dissociation of coactivator PGC-1α and silencing of the ERRγ target gene PDK4 [10], [25]. As demonstrated in Figure 6, we observed that ERRγ specific inverse agonist GSK5182 significantly reduces the hypoxia-induced expression of PDK4. These results show that GSK5182 is important pharmacological regulator of the expression of PDK4 under hypoxia. Recent reports demonstrate that chronic alcohol consumption results in hepatic hypoxia and steatosis. The increase in HIF-1α caused by chronic alcohol feeding accelerates lipid accumulation in hepatocytes [26]. Chuvash polycythemia, an autosomal recessive human disorder, shows high levels of HIF at normal oxygen tensions by impaired regulatory degradation of HIF1. Patients with Chuvash polycythemia express high levels of PDKs in skeletal muscle and have high levels of lactate and pyruvate in their blood [27]. In addition, obstructive sleep apnea, results in chronic intermittent hypoxia and increased HIF-1α protein expression [28]. These maladaptive responses have pathological consequences that include liver damage. Our finding that PDK4 expression is suppressed by GSK5182 may have therapeutic potential for the treatment of conditions that induce intermittent hypoxia.

In a summary, as depicted in Figure 6E, we propose that hypoxia induces ERRγ gene expression via activation of HIF-1α. The transcriptional activation activity of the resulting ERRγ protein on the PDK4 promoter is further stimulated by an association with HIF-1α. Our findings suggest that the regulation of transcriptional activity of ERRγ by its specific inverse agonist may prove useful for the regulation of hypoxia-mediated PDK4 gene expression.

## Materials and Methods

### Chemicals

Desferrioxamine (DFO) was purchased (Invitrogen). ERRγ inverse agonist GSK5182 was synthesized according the method described [13]. GSK5182 were dissolved in DMSO.

### Plasmid and DNA construction

The plasmids of pCMV-β-gal and pcDNA3-ERRγ were described elsewhere [29], [30]. pcDNA3-HIF-1α, pcDNA3-ARNT were kindly provided by Dr.Eric Huang. The reporter PDK4-Luc was kind gifts from Drs. Dieter Kressler [31], Akiyoshi Fukamizu [32] and Robert A. Harris [33], respecively. Human ERRγ deletion constructs, pGL3-hERRγ (−1 kb), pGL3-hERRγ (−0.5 kb) and pGL3-hERRγ (−0.3 kb), were subcloned via the insertion of the PCR fragments of human ERRγ promoter into pGL3 between MluI and XhoI sites. Human PDK4 deletion constructs, pGL3-hPDK4 (−841 bp), pGL3-hPDK4 (−500 bp) and pGL3-hPDK4 (−291 bp), were constructed by insertion of the PCR fragments of human PDK4 promoter into pGL3 between NheI and XhoI sites. The mutant reporters of hERRγ-mtHRE1-Luc, hERRγ-mtHRE2-Luc, hERRγ-mtHRE1+2-Luc and hPDK4-mtERRE1-Luc were generated with the Quickchange site-directed mutagenesis kit (Stratagene). All plasmids were confirmed via sequencing analysis. All plasmids were confirmed via sequencing analysis.

### Cell Culture and hypoxia treatment

HepG2 cells were maintained in DMEM (invitrogen Carlsbad,CA), supplemented with 10% fetal bovine serum(FBS; Cambrex Bioscience Walkersville, Inc., Walkersville, MD) and antibiotics (Invitrogen, Carlsbad, CA). Human hepatoma HepG2 cells were purchased from American Type Culture Collection (ATCC HB-8065). Cells were exposed to hypoxia, 0.1% O_2_, by incubating cells at 37°C in 5% CO_2_/10% H_2_/85% N_2_ anacrobic incubator (Forma Scientific). Hypoxia was also induced chemically by treating cells with DFO.

### Transient Transfection Assay

Cells were split in 24-well plates at desities of 2–8×10^4^cells/well the day before transfection. Transient transfections were performed using the Lipofectamine™ 2000 reagent (Invitrogen) according to the manufacturer's instruction. Cells were transfected with expression vectors, a reporter gene, and the control *lacZ* expression plasmid pCMVβ. Total DNA amount was kept constant by adding the pcDNA3 empty vector. Cells were harvested approximately 40–48 h after the transfection for luciferase and β-galactosidase assays. The luciferase activity was normalized with β-galactosidase activity. Fold activity was calculated considering the activity of reporter gene alone as 1. The data is representative of at least three to five independent experiments.

### Recombinant Adenovirus

Adenoviruses expressing, unspecific (US) shRNA were previously descrived [34]. Adenoviruses expressing shERRγ were generated with the pAd-easy system as described [34]. All viruses were purified by using CsCl or Adeno-X™ Maxi Purification Kit (Clontech).

### RNA interference

HepG2 cells were transfected with siRNA using Lipofectamin 2000 (Invitrogen) reagent according to the mafacturer's protocol. Forty-eight hours after transfection, total protein was isolated for Western blot for HIF-1α or ERRγ and β-tubulin or α-tubulin as a control. The sequences of siRNA are as follows: siHIF-1α, sense 5′-CCUAUAUCCCAAUGGAUGAUGTT-3′, siERRγ, sense 5′-UGGCCAUCAGAACGGACUU-3′ and control nonspecific siRNA.

### RNA isolation and Realtime PCR analysis

Total RNAs were extracted under various conditions using TRIzol reagent (Invitrogen) according to manufacturer's protocol. The mRNAs of ERRγ, PDK2, PDK4, VEGF and L32 were analyzed by qPCR as indicated. PCR was performed in the following PCR condition of denaturating at 94°C for 30 seconds, annealing at 60°C for 30 seconds and elongation at 72°C for 30 seconds. All data was normalized to ribosomal L32 expression or β-actin. DNA samples from total RNA reverse transcription served as the templates for qPCR experiments, which were performed with Power SYBR Green PCR Master Mix (Applied Biosystems, Carlsbad, CA) and the Apploed Biosystems StepOnePlus™ real-time PCR system (Applied Biosystems) in triplicate. The sequences of siRNA are as follows : ERRγ, forward primer 5′-CTCCAGCACCATCGTAGAGGATC-3′, reverse primer 5′-GATCTCACATTCATTCGTGGCTC-3′, VEGF, forward primer 5′-TCCACCCTGCCAAGTGGTCC-3′, reverse primer 5′-AGGAAGCTCATCTCTCCTAT-3′, PDK2, forward primer 5′-GAAGAATGCGTCCCTGGCAG-3′, reverse primer 5′-GGTTCCGGATGGTGACCAGG-3′, PDK4, forward primer 5′-CCC GCTGTCCATGAAGCAGC-3′, reverse primer 5′-CCAATGTGGCTTGGGTTTCC-3′

### Western blot analysis

About 80% confluences of HepG2 cells were treated with DFO and Cells were harvested with RIPA cell lysis buffer (Elpis-Biotech). Proteins from whole cell lysates were separated on 10% SDS-PAGE and then transferred to nitrocellulose membranes (Sigma-Aldrich). The membrane were probed with monoclonal ERRγ antibodies (R&D Systems), HIF-1α (Santa Cruz), β-tubulin and α-tubulin, respectively and then visualized using an ECL kit (Amercham Bioscience), according to the manufacturer's instructions.

### Chromatin Immunoprecipitation (ChIP) assay

ChIP assay was performed as previously described [10]. In brief, hypoxia treated HepG2 cells in 100-mm culture dishes were fixed with 1% formaldehyde, washed with ice-cold PBS, harvested and solicited. The soluble chromatin was then subjected to immunoprecipitation using anti-HIF-1 α (Santa Cruz) or anti-ERRγ (R&D Systems) antibodies followed by using protein A agarose/salmon sperm DNA (upstate). IgG was used a negative control for immunoprecipitation. Precipitated DNA was recovered via phenol/chloroform extraction and amplified by RT-PCR for 30–35 cycles using specific primer sets for the indicated specific promoter regions of ERRγ and HIF-1α genes. The sequences of siRNA are as follows : HIF-1α (−1826∼−1856 positions targeting), forward primer 5′-GCTTTTATACAGTATTCTGAGGC-3′, reverse primer 5′-ATTTTCTAGGTCTTTAAAAAACCAA-3′, HIF-1α (−1080∼−849 positions targeting), forward primer 5′-TTATGTATAGAAACAAATGTGTACA-3′, reverse primer 5′-ATGCATTTTGACAAAATAAAGACAG-3′, HIF-1α (−508∼−295 positions targeting), forward primer 5′-GACATGATTAGGAATACATGAGAA-3′, reverse primer 5′-ACAAAAGAAACATAACTAACTTAAC-3′, ERRγ (−1056∼−886 positions targeting), forward primer 5′-GGAATCACAAAGCTGCATCA-3′, reverse primer 5′-GCTGCAAAAGGTCTTCCTTG-3′, ERRγ (−502∼−252 positions targeting), forward primer 5′-AGAAGCCCATACTTTTGAAC-3′, reverse primer 5′-AGAGGAAGCAGAAACAGGTA-3′

### Statistical analysis

Data were expressed as means ± S.E.M. Statistical analysis was conducted via Student's *t* test. Differences were considered statistically significant at *p*<0.05.
